# Short-chain perfluoroalkyl acids: environmental concerns and a regulatory strategy under REACH

**DOI:** 10.1186/s12302-018-0134-4

**Published:** 2018-02-27

**Authors:** Stephan Brendel, Éva Fetter, Claudia Staude, Lena Vierke, Annegret Biegel-Engler

**Affiliations:** German Environment Agency, Wörlitzer Platz 1, 06844 Dessau-Roßlau, Germany

**Keywords:** REACH, SVHC, PFASs, Candidate list, Restriction, Regulation, Per- and polyfluoroalkyl substances, PFAAs

## Abstract

**Background:**

Short-chain PFASs (per- and polyfluoroalkyl substances) are widely used as alternatives to long-chain PFASs. Long-chain PFASs become gradually regulated under REACH (EC No. 1907/2006) and other international regulations, due to having persistent, bioaccumulative and toxic properties and/or being toxic for reproduction. The increasingly used short-chain PFASs are assumed to have a lower bioaccumulation potential. Nonetheless, they have other properties of concern and are already widely distributed in the environment, also in remote regions. The REACH Regulation does not directly address these emerging properties of concern, complicating the implementation of regulatory measures. Therefore, this study illustrates these environmental concerns and provides a strategy for a regulation of short-chain PFASs within REACH.

**Results:**

Short-chain PFASs have a high mobility in soil and water, and final degradation products are extremely persistent. This results in a fast distribution to water resources, and consequently, also to a contamination of drinking water resources. Once emitted, short-chain PFASs remain in the environment. A lack of appropriate water treatment technologies results in everlasting background concentrations in the environment, and thus, organisms are permanently and poorly reversibly exposed. Considering such permanent exposure, it is very difficult to estimate long-term adverse effects in organisms. Short-chain PFASs enrich in edible parts of plants and the accumulation in food chains is unknown. Regarding these concerns and uncertainties, especially with respect to the precautionary principle, short-chain PFASs are of equivalent concern to PBT substances. Therefore, they should be identified as substances of very high concern (SVHC) under REACH. The SVHC identification should be followed by a restriction under REACH, which is the most efficient way to minimize the environmental and human exposure of short-chain PFASs in the European Union.

**Conclusion:**

Due to an increasing use of short-chain PFASs, an effective regulation is urgently needed. The concerns of short-chain PFASs do not match the “classical” concerns as defined under REACH, but are not of minor concern. Therefore, it is of advantage to clearly define the concerns of short-chain PFASs. This might facilitate the following restriction process under REACH.

## Background

Some representatives of per- and polyfluoroalkyl substances (PFASs) are highly stable organic compounds, due to the C–F bond being one of the strongest in organic chemistry [1]. Compared to hydrocarbons, PFASs have enhanced technical properties, such as a higher surface activity and better dielectric properties, and possess at the same time a higher thermal stability, an increased chemical resistance and a physiological inertness [2]. Due to these properties, PFASs are well suited for manifold applications and have been used extensively in various industrial and consumer applications since the 1950s, e.g., impregnation of textiles, paper, hard metal plating, in paints, the production of fluoropolymers, etc. [3, 4]. During the production and life cycle of the products, a certain fraction of PFASs is inevitably emitted into the environment (e.g., [5]). Many commonly used PFASs can degrade under environmentally relevant conditions to perfluoroalkyl acids (PFAAs), regarded as final degradation products (e.g., [6]). Besides the direct emission of PFAAs, e.g., from production and life cycle of certain fluoropolymers, indirect emissions via degradation of the precursor substances are of high relevance regarding the global emissions [7]. Also, side-chain fluorinated polymers, in particular the group of side-chain fluorotelomer-based polymers, were shown to be degraded to PFAAs under environmental conditions in the range of decades to centuries [8] and are therefore considered to be precursors. As highly persistent final degradation products of many other PFASs, PFAAs are a relevant group regarding environmental and human exposure, even though being a small substance group within the whole PFASs group, consisting of at least 3000 substances [9].

The concern of possible risks of PFAAs to the environment and human health was broadly unrecognized until the turn of the millennium, when two groups of PFAAs, long-chain perfluoroalkyl carboxylic acids and long-chain perfluoroalkane sulfonic acids, have been shown to be ubiquitously present in biota and humans (e.g., [10, 11]). Since becoming aware of the ubiquitous distribution and the hazardous properties (see [12]) of long-chain PFASs (i.e., long-chain PFAAs and their precursors), voluntary measures of manufacturers and regulatory action have been taken place to reduce the emissions. Perfluorooctane sulfonic acid (PFOS), its salts and perfluorooctane sulfonyl fluoride (PFOSF) are listed as persistent organic pollutant (POP) under the Stockholm Convention and perfluorooctanoic acid (PFOA) and related precursors are proposed as POP. Under the European Chemicals Regulation (REACH EC No. 1907/2006), several long-chain perfluoroalkyl carboxylic acids (C_8_–C_14_) and the long-chain perfluorohexane sulfonic acid are identified as substances of very high concern (SVHC) and included in the REACH candidate list. In the EU restriction, proposals for the perfluoroalkyl carboxylic acids on the candidate list are in progress, but only implemented for PFOA its salts and related substances up to the present. In summary, global measures to ban long-chain PFAAs of concern are ongoing and need to be continued.

**Fig. 1:**
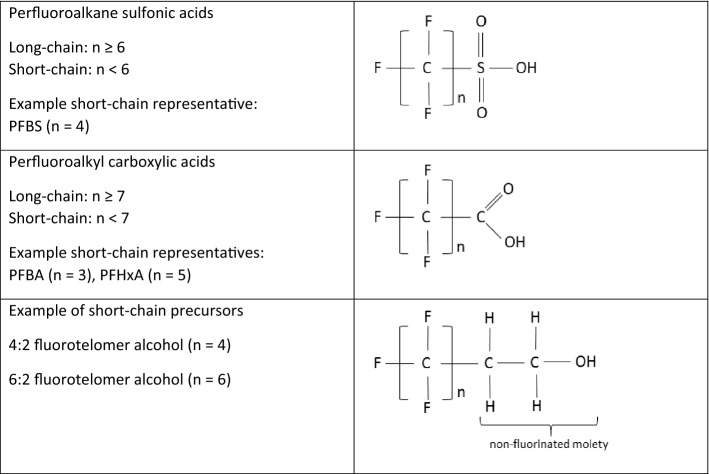
Terminology of per- and polyfluoroalkyl substances

With the regulation of long-chain PFASs and due to voluntary measures, non-regulated PFASs have been more extensively used as alternatives. Information of these replacements are not easily accessible, however, it is known that short-chain PFASs (i.e., short-chain PFAAs and respective precursors, see Fig. 1 for terminology) are important and broadly used alternatives [13]. Due to historical emissions mainly as unintended by-products during the manufacturing of long chain PFASs in the past, short-chain PFASs are already present in environmental compartments [7, 14]. The replacement of long-chain PFASs with short-chain PFASs and in general other PFASs is highly criticized [15–17], as short-chain PFASs have also relevant properties of concern.

Long- and short-chain PFAAs were originally distinguished, since short-chain PFAAs are assumed to have a lower bioaccumulation potential and improved environmental properties compared to long-chain PFAAs [3, 18]. However, short-chain PFAAs are as persistent as long-chain PFAAs have different, but not less alarming properties of concern, and are already widely distributed in the environment [e.g., 19–27]. The awareness of these concerns needs to be raised to obtain a fast regulation of short-chain PFASs. Therefore, this study (i) summarizes and highlights the concerns of short-chain PFAAs, (ii) investigates the need for a regulation and (iii) provides a strategy for how a regulation could be implemented in REACH.

## Results and discussion

### Uses and sources of environmental exposure

According to the publicly available information on REACH registrations, short-chain PFAAs, e.g., PFBA (perfluorobutanoic acid), PFBS (perfluorobutanesulfonic acid) and PFHxA (perfluorohexanoic acid) have not been registered so far. The last registration deadline under REACH for substances which are manufactured or imported between one and 100 tons per year is 31 May 2018. However, registration obligations do not include substances in imported articles. In contrast to the short-chain PFAAs, various short-chain precursor substances are registered in high tonnages (> 100 tons per annum) and are mainly used for manufacturing polymers. Those polymers may most likely have wide dispersive uses. In general, short-chain PFASs can substitute long-chain PFASs in manifold applications [28]. Therefore, they are used comparably to long-chain PFASs in many consumer products and industrial applications requiring inert or repellent characteristics. Due to a partially limited technical performance in comparison with long-chain PFASs, higher amounts of short-chain PFASs are usually used in compensation (e.g., in wax and polish) [28]. During the production and life cycle of the products, inevitably releases of short-chain PFASs into the aquatic and terrestrial environment occur [29]. There are vast amounts of applications, but only scarce information on uses of short-chain PFASs. Additionally, the degradation of side-chain fluorinated polymers might contribute significantly to emissions of short-chain PFASs in the long-term, as demonstrated by an environmental fate modeling analysis by Li et al. [30]. Besides degradation of the polymers in the long-term, non-bound residues can easily reach the environment and may thus contribute to future releases [29, 30]. Due to the hundreds of various precursors, assessing the uses of all precursors is nearly impossible. The Swedish Chemicals Agency [4], the Norwegian Environment Agency [31] and the Danish Ministry of the Environment [32] provided detailed information on the use of short-chain PFASs to the extent possible. In the following, information for some use areas is summarized.

In recent years, surfactants in foam-based fire-fighting agents, used to extinguish flammable liquids or gases, shifted from long-chain PFASs (mainly C8-based) to short-chain PFASs (mainly C6-based). The stability and surface-active properties of PFASs enable a film formation on top of burning liquids, extinguishing the fire and preventing burn back. Nowadays, a number of foam-based fire-fighting agents contain short-chain PFASs. The use of fire-fighting agents results in direct contaminations of the environment when there is no possibility of collecting the fire water and subsequent disposal [33, 34].

Textiles, leather, carpet, apparel, and upholstery are desired to be oil, water and dirt repellent. For some papers and food contact materials, greaseproof properties are required. Therefore, these items can be impregnated with side-chain fluorinated polymers, consisting of non-fluorinated carbon backbones connected to short-chain PFAS side-chains such as fluorotelomer alcohols.

Short-chain PFASs are furthermore used for example as mist suppressing agents in hard metal-plating [35], in the electronic industry due to insulating and water repellent properties [36], in cosmetic products to make the products oil and water repellent [37], in paints and inks to improve wetting, smoothness and flow [36], in medical devices [4] and the oil production [32].

### Overview of concerns of short-chain PFAAs

This section specifies concerns attributed in general to short-chain PFAAs. The properties of concerns only attributed to certain PFAAs or precursor substances are not addressed. However, precursors share the common concern of degradation to short-chain PFAAs as final degradation products. It is therefore possible to include precursors in a regulation of short-chain PFAAs (see “Strategy for regulation of short-chain PFASs under REACH”).

#### Short-chain PFAAs are extremely persistent

Based on the high energy of the carbon–fluorine bond [2], it can be assumed that short-chain PFAAs are extremely persistent similar to the persistence of long-chain PFAAs [38, 39]. They do not undergo abiotic or biotic degradation at all under environmental conditions and are considered highly stable transformation products in which several precursors ultimately degrade into [13, 40]. This extreme persistence is regarded as an incalculable hazard itself, as short-chain PFAAs will stay in the environment for decades to centuries [41]. The possible risks of such extremely persistent organic fluorochemicals for humans and the environment have been emphasized by leading scientists [16, 17]. Each released PFAS molecule remains in the environment, meaning that it is impossible to reverse exposure. Thus, levels of these substances will most probably increase over time in certain compartments. As a consequence, long-term effects on humans and wildlife may be possible in the future, when certain effect thresholds are reached.

#### Short-chain PFAAs have a low adsorption potential and are very mobile

The determination of the physicochemical properties of short-chain PFAAs requires, due to the unusual substance properties, specific methods. Estimates of the physicochemical properties are available (PFBS, PFBA and PFHxA [42, 43]: log*K*_ow_ (neutral form) = 2.82–4.6, water solubility > 20 g/L, log*K*_oa_ = 6–6.7, pK_a_ < 1, log*K*_oc_: 2.7–3.6), showing that short-chain PFAAs are very mobile, which is also confirmed by their environmental distribution [44, 45]. Due to this mobility short-chain PFAAs effectively reach water bodies which is of special concern regarding human exposure: Drinking water resources are highly sensitive to contamination with short-chain PFAAs [19, 46]. Due to the low adsorption potential, short-chain PFAAs will not bind to particles and stay mainly dissolved in the water phase.

#### In a larger scale, once emitted short-chain PFAAs can only hardly be removed from water

Long-chain PFAAs can be removed from water with activated carbon filters. However, this is not as effective for short-chain PFAAs due to the low adsorption potential [47]. Hence, at a large scale short-chain PFAAs can only hardly, if at all, be removed from the environment with the main methods available today [46, 48, 49]. Some promising methods are very costly and just applied at a laboratory scale [50]. The absence of effective measures on a larger scale is problematic in particular with regard to contaminated drinking water reservoirs. Since short-chain PFAAs are not expected to degrade chemically and biologically, the concentrations in contaminated areas will only decline with a further spatial distribution, provided that no further release occurs.

#### Short-chain PFAAs have the potential for long-range transport. Already today, short-chain PFAAs are monitored in remote regions and show a wide spread distribution in biotic and abiotic compartments

Due to the high mobility caused by the high aqueous solubility and relatively low adsorption potential of short-chain PFAAs, they have a higher potential for long-range transport compared to the long-chain homologues [43]. Additionally, volatile precursors are likely to be transported to remote regions via the atmosphere and then being degraded to short-chain PFAAs. Monitoring data show that already today short-chain PFAAs are present in remote areas [20, 51, 52] and have a wide spread distribution in biotic and abiotic compartments. Table 1 shows examples of monitoring data from Europe. In a few cases, increasing concentrations of short-chain PFAAs in the environment and biota are already observed; for example, for PFBS in dolphins from the South China Sea [53], for PFHxA in water samples near a fluoropolymer production plant in Japan [54] and for PFBA in snow from remote regions in the European Alps [25, 52]. As demonstrated in a recent review by Land et al. [55], a generalized prediction of temporal trends in environmental concentrations of short-chain PFAAs is currently not feasible due to limited monitoring data and insignificant time trends shown in most of the studies. Historical releases of short chain PFASs are hard to quantify [7]. Because the substances are not degradable, historical releases, i.e., as unintended by-products during the manufacturing of long chain PFASs contributed to the environmental stocks to a reasonable amount and impede the observation of significant trends. However, due to the phase-out of long-chain PFASs [18, 56], manufacturing and use of short-chain PFAAs and related substances are very likely to further increase in the near future. Thus, emissions of short-chain PFAAs will increase as well. Along with the expected increasing emissions, short-chain PFAAs will further enrich in the environment leading to increased background concentration levels, in the long-term especially in the aquatic systems [57].

**Table 1:** Example monitoring data from Europe regarding PFBS, PFBA and PFHxA

Sampling	Short-chain PFAA and concentration^a^	Reference/sampling year
Tab water [ng/L]		
France, 8 locations	PFBS: 3.2 (32%);	[19]
	PFHxA: nd	Sampling year: 2014
Germany, 26 locations	PFBS: 2.7 (42%)	[21]
	PFHxA: 2 (23%)	
	PFBA 2 (19%)	
Spain, 84 locations	PFBA: 10 (52%)	[22]
	PFHxA: 4.7 (18%)	Sampling year: 2010–2011
	PFBS: 8.3 (35%)	
Surface water [ng/L]		
Spain, Ebro	PFBA: 35.2 (58%)	[23]
	PFHxA: 1.7 (8%)	Sampling year: 2010
	PFBS: nd	
Spain, Guadalquivir	PFBA: 214.3 (92%)	
	PFHxA: nd	
	PFBS: 10.1 (8%)	
Germany, Elbe	PFBA: 2.6 (100%)	[24]
	PFBS: 7.5 (100%)	Sampling year: 2015
	PFHxA: 1.5 (100%)	
Germany, Saale	PFBA: 0.5 (100%)	
	PFBS: 4.3 (100%)	
	PFHxA: 4 (100%)	
Snow [ng/L]		
European Alps	PFBA: 0.69	[25]
	PFHxA: 0.06	Sampling year: 2008
Sediments [ng/g dw]		
Baltic sea	PFBS: 0.00017 (75%)	[26]
	PFHxA: 0.115 (100%)	Sampling year: 2013–2014
Organisms [ng/g ww]		
Baltic sea		[26]
Zooplankton	PFBS/PFHxA: nd	Sampling year: 2013–2014
Herring	PFBS/PFHxA: nd	
Sprat	PFBS/PFHxA: nd	
Guillemot egg	PFBS: 0.0035 (100%)	
	PFHxA: 0.0026 (50%)	
Ingolstadt, Germany		[27]
Wild boar	PFHxA: 0.49 (66%)	Sampling year: 2011–2012
	PFBA: 0.73 (100%)	
	PFBS: 0.25 (53%)	
Svalbard, Norway		[20]
Reindeer	PFBA: 0.42 (56%)	
Ebro, Spain		[23]
Fish	PFBS: 4.9 (69%)	Sampling year: 2010
	PFBA: 0.6 (31%)	
	PFHxA: 268.4 (56%)	

^a^Mean values, including frequency detected

#### The permanent exposure to short-chain PFAAs results in continuous and poorly reversible concentrations in organisms. There is a risk of adverse effects on humans and the environment, which will rise with increasing exposure

Considering that the exposure to short-chain PFAAs is unlikely to be stopped shortly, there will be increasing continuous and poorly reversible environmental background concentrations of short-chain PFAAs. Consequently, organisms and humans will be permanently exposed to short-chain PFAAs, resulting in continuous and poorly reversible internal concentrations. The poorly reversible internal concentrations in organisms are caused by the persistence of short-chain PFAAs and their continuous presence in the environment. Therefore, the organismal elimination efficiencies are of secondary relevance. This becomes more evident when considering that the half-lives of short-chain PFAAs in the environment exceed their half-lives in the organisms by far. Cousins et al. [41] addresses this approach in more detail, showing exemplary that the contamination of drinking water resources with short-chain PFAAs leads to a poorly reversible exposure in humans, comparably to a contamination with long-chain PFAAs. Due to the high mobility of short-chain PFAAs and the missing suitable water treatment technologies for drinking water, short-chain PFAAs will remain in the water resources over time (this scenario proves true, see “Case example Rastatt”). This results in a permanent exposure via drinking water, independently of the elimination half-lives of short-chain PFAAs.

Nevertheless, elimination half-lives of short-chain PFAAs are non-negligible: Depending on the species, they range from a couple of hours to several days in some mammals [58–60], with longer half-lives in humans (e.g., 30 days for PFHxA [61]). The elimination half-lives are shorter compared to some long-chain homologues, but nevertheless of concern, as short-chain PFAAs will still remain in organisms with continuous exposure. Some short-chain PFAAs have considerable protein binding potentials [62, 63]. A high protein binding potential is of toxicokinetical relevance: the blood can easily distribute short-chain PFAAs within the body resulting in a potential to enrich particularly in blood rich tissues. Data from the exposure of mammals show that short-chain PFAAs can be widely distributed in the organism [64, 65].

Possible adverse effects, especially long-term effects, caused by a high protein binding potential are uncertain. In general, there is a high level of uncertainty, if the permanent exposure with low concentrations of short-chain PFAAs may cause adverse effects in organisms. There is insufficient data on the toxic properties of short-chain PFAAs [66], and therefore, sub-lethal long-term effects cannot be excluded. There might be unconsidered sensible organisms, certain development stages or effects on multiple generations, being not considered when assessing the toxicity of short-chain PFAAs. Mixture toxicity of different PFASs or the toxicity of PFASs in combination with natural and anthropogenic stressors is largely unknown [57].

#### The distribution of short-chain PFAAs in terrestrial systems and along food chains is unknown. Short-chain PFAAs are known to enrich in edible parts of plants

Short-chain PFAAs are known to enrich in plants and due to their high water solubility and low adsorption potential, especially in leaves and fruits [67–69]. This enrichment in the edible parts of plants is higher compared to long-chain PFAAs and might pose a risk regarding a distribution along the food chain, which has not yet been investigated. In contaminated soil, especially in former arable land, the accumulation in the edible parts of plants is of concern due to human exposure (exemplary shown in “Case example Rastatt”). Field studies do not allow drawing conclusions on distributions of short-chain PFAAs along the food chains. Especially, the accumulation in terrestrial organisms, as shown for long-chain PFAAs [70], cannot be excluded [71].

### Case example Rastatt

In the surroundings of Rastatt (Baden-Wuerttemberg, Germany), 480 hectares of former arable land are contaminated with short-chain PFAAs and precursors. The pollution was detected in 2013 and has probably been caused by the longstanding application of compost mixed with sludge from paper production, contaminated with various precursors. Over time, the precursors contained in the soil degraded to short-chain PFAAs and were enriched in plants. Local authorities derived thresholds for short-chain PFAAs in food (in the µg/kg range) [72], based on guidance values for drinking water [73]. Pre-harvest monitoring showed that the concentration of short-chain PFAAs in some crops exceeded these thresholds, preventing the use as food. Crops enriching high amounts of short-chain PFASs are recommended not to be cultivated for consumption on these contaminated fields (e.g.,asparaguses, strawberries and other vegetables). Despite the recommendations for cultivation, threshold values are in parts exceeded to date. This might be due to a strong influence of insufficiently predictable abiotic factors, such as organic carbon content, on the enrichment of short-chain PFASs in plants [74]. Over time, the very mobile short-chain PFASs and precursors in the soil also wash out into the groundwater. Irrigating crops with contaminated water are causing further emissions to soil and uptake into plants. Two groundwater wells for drinking water production had to be closed [72].

For several years, the dimension of the contamination and the problems arising with the contamination of short-chain PFASs has not been recognized. Estimations addressing all adverse environmental effects and socio-economic costs resulting from the contamination are not available, but according to information given by the water work solely the cost for water treatment with charcoal filters amounts to several million Euros. Until now, no practicable solution for removing the short-chain PFASs from the soil or groundwater has been found. Effective solutions, such as a removal and replacement of the top soil or “pump and treat” methods, would be far too expensive. Furthermore, there are still high concentrations of short-chain PFASs and unknown precursors contained in the soil and there is no applicable solution for stopping them from reaching the groundwater. Research to find an appropriate solution is ongoing.

This Rastatt case clearly shows that once emitted to the environment, short-chain PFAAs cause irreversible contaminations, and thus causing high socio-economic costs and threats to man and environment. Besides Rastatt, there is a large scale contamination of drinking water with short-chain PFASs in Uppsala known. The contamination in Sweden is mainly caused by the use of aqueous film forming fire-fighting foams at a military airport [33].

### Strategy for regulation of short-chain PFASs under REACH

PFAAs themselves are not produced in high tonnages. Primarily manifold precursor substances degrading in the environment to PFAAs are responsible for their emission to the environment and subsequently to humans. Those precursor substances are defined by a similar length of the perfluorinated carbon chain compared to the short-chain PFASs, connected to a non-fluorinated moiety (see Fig. 1).

A regulation of every single precursor is impracticable due to the unknown but likely vast amount of precursors on the market. For an effective regulatory measure, it is mandatory to address both PFAAs and their precursor substances, considering also emissions from imported articles. These considerations have already been outlined by Vierke et al. [39] regarding the long-chain perfluoroalkyl carboxylic acid PFOA. Precursors registered under REACH are mainly used for manufacturing polymers according to industry. Those polymers (i.e., side-chain fluorinated polymers) contain unbound residues of PFASs precursors or the polymers may breakdown over time and release PFAS precursors [8]. Thus, polymers are included in such a regulation as well.

In short, as a first step identification as SVHC according to Article 57 of the REACH regulation needs to be considered. Such substances become included in the REACH candidate list. Article 57 of the REACH Regulation allows to include substances on the candidate list that (i) are classified as carcinogenic, mutagenic or toxic for reproduction (CMR) category 1A or 1B, (ii) have persistent, bioaccumulative and toxic (PBT) or (iii) very persistent and very bioaccumulative (vPvB) properties according to REACH (Annex XIII), or (iv) have an equivalent level of concern as CMR or PBT/vPvB substances. The inclusion of a substance in the candidate list results in obligations for the manufacturer to provide information to downstream users and to consumers (if > 0.1% proportion in articles). This regulatory measure alone is not sufficient to control the emissions of short-chain PFASs. But the inclusion into the candidate list is a strong signal itself since it contains all substances of very high concern identified within the EU. For substances on the candidate list REACH foresees authorisation as the regulatory measure. In such case, using the substance is only allowed if authorization was granted. However, an authorisation does apply only for the substance itself and does not include any precursor substances. Furthermore, the authorisation procedure does not include imported articles, probably contributing to the emissions of short-chain PFASs in Europe. Consequently, starting the authorisation procedure would not constitute an effective risk management option. A restriction (Article 67) can cover the placing of a substance on the market, the content of a substance in (imported) articles, the use of such a substance on its own or in a mixture and respective precursors. Thus, a general restriction can address all relevant emission routes and is considered the appropriate regulatory measure for short-chain PFASs under REACH. To decide if certain exemptions might be appropriate, further detailed information on uses and alternatives are necessary. Once in force, the restriction is valid for the European Economic Area (EEA), having a population of more than 500 million people.

### The equivalent level of concern

With the current knowledge, short-chain PFASs seem not to fulfill the bioaccumulation or toxicity criteria of REACH Annex XIII. Due to the special properties of short-chain PFASs, they might not fulfill the “classical” properties of concern of SVHCs, being clearly defined within Article 57(d and e) of the REACH Regulation. However, given the properties of concern (see “Overview of concerns of short-chain PFAAs”) and the already existing pollutions (see “Case example Rastatt”), there is an unequivocal need for regulation of short-chain PFASs. Within the REACH Regulation, it is possible to identify substances as SVHC that have an equivalent level of concern compared to CMR or PBT/vPvB substances, as defined in article 57(f) (see “Strategy for regulation of short-chain PFASs under REACH”). So far, the equivalent level of concern approach is applied for endocrine disrupting chemicals, sensitisers and specific target organ toxicity after repeated exposure only. However, in our opinion short-chain PFASs are also of equivalent level of concern compared to PBT/vPvB substances and can be identified as SVHC for the following reasons:

Article 1 of the REACH Regulation states that the provisions of the REACH Regulation are underpinned by the precautionary principle. The realization of the precautionary principle is apparent when looking at the vPvB criteria for the SVHC identification (Article 57(e)). Even though a substance does not cause any toxic effect, it can be identified as SVHC because it is due to its intrinsic properties expected to remain in the environment and to accumulate in organisms. The reasoning is that (i) the level of uncertainty in identifying long-term effects cannot be estimated with sufficient accuracy (vB) and (ii) consequences of an underestimation of adverse effects are not easily reversible by regulatory action (vP), i.e., the effect is occurring or is likely to occur at a certain point in time, and even if there is immediate regulatory action to prevent further emission, the adverse effects will continue. The extreme persistence and other intrinsic properties (e.g., low adsorption potential) of short-chain PFASs unquestionable fulfill the second concern. In practice, this is shown in the “case example Rastatt”. The first issue, that long-term effects cannot be estimated with sufficient accuracy, is in case of vPvB substances met by the very high bioaccumulation potential. Short-chain PFASs do not fulfill the criteria according to Annex XIII of the REACH Regulation for being bioaccumulative. However, as they will not degrade in the environment, there is a permanent and irreversible exposure of organisms. With ongoing emission, the environmental concentrations of short-chain PFASs will most probably increase, due to the lack of degradation and the fact that emerging emissions come on top of already released amounts resulting in an accumulation in certain environmental compartments. There is a high uncertainty about the toxicity of short-chain PFASs, especially regarding long-term effects (see “Overview of concerns of short-chain PFAAs”). If emissions continue and environmental concentrations of short-chain PFASs exceed a certain limit, it seems very likely that adverse effects may occur. Even if the releases of short-chain PFASs will stop immediately, the already emitted short-chain PFASs do irreversibly remain in the environment (see “Case example Rastatt”). These considerations and the additional concerns, such as enrichment in the edible parts of plants and the accumulation in water resources (see “Overview of concerns of short-chain PFAAs”), show that short-chain PFASs are of equivalent level of concern compared to PBT/vPvB substances.

## Conclusion

Short-chain PFASs are increasingly used and are considered to have properties of very high concern according to Article 57(f) of the REACH Regulation: They are of equivalent level of concern to PBT or vPvB substances. The outlined concerns related to short-chain PFASs, the already existing pollution, i.e., locally restricted very high pollutions (e.g., Rastatt and Uppsala) and global pollutions due to historical emissions, and very likely further increasing concentrations in the environment and biota illustrate that a fast regulation is mandatory. In accordance with the regulation of long-chain PFASs, the most effective way constitutes an SVHC identification of the short-chain PFAAs followed by a restriction, allowing also the regulation of precursors. The SVHC identification is not a prerequisite but will be advantageous, as the concerns attributed to short-chain PFASs do not comply with the “classical” concerns considered in the REACH Regulation and need to be clearly defined. In addition to a regulation by authorities, voluntary measures by industry could contribute substantially in reducing the emissions of short-chain PFASs.

## Methods

A literature review was conducted to obtain available information on short-chain PFASs. The concerns of short-chain PFASs and its equivalent level of concern with regard to the REACH Regulation were discussed and the concern was specified at a workshop in Berlin, organized by the German Environment Agency (UBA) in October 2016. Regulatory experts and scientists from different EEA Member States, the European Commission, the European Chemicals Agency, USA and Australia participated.

## Abbreviations

CMR: carcinogenic, mutagenic or toxic for reproduction
EEA: European Economic Area
K_OA_: octanol–air partition coefficient
K_OC_: organic carbon–water partition coefficient
K_OW_: octanol–water partition coefficient
PBT: persistent, bioaccumulative and toxic
PFAA: perfluoroalkyl acid
PFASs: per- and polyfluoroalkyl substances
PFBA: perfluorobutanoic acid
PFBS: perfluorobutane sulfonic acid
PFHxA: perfluorohexanoic acid
PFOA: perfluorooctanoic
PFOS: perfluorooctane sulfonic acid
pK_a_: acid dissociation constant
POP: persistent organic pollutant
REACH: European Chemicals Regulation, EC No. 1907/2006, Registration, Evaluation and Authorization of Chemicals
SVHC: substance of very high concern
vPvB: very persistent and very bioaccumulative
